# Wolf Lethal Control and Livestock Depredations: Counter-Evidence from Respecified Models

**DOI:** 10.1371/journal.pone.0148743

**Published:** 2016-02-11

**Authors:** Niraj Poudyal, Nabin Baral, Stanley T. Asah

**Affiliations:** 1 School of Arts, Kathmandu University, Lalitpur, Nepal; 2 School of Environmental and Forest Sciences, College of the Environment, University of Washington, Seattle, Washington, United States of America; Michigan Technological University, UNITED STATES

## Abstract

We replicated the study conducted by Wielgus and Peebles (2014) on the effect of wolf mortality on livestock depredations in Montana, Wyoming and Idaho states in the US. Their best models were found to be misspecified due to the omission of the time index and incorrect functional form. When we respecified the models, this replication failed to confirm the magnitude, direction and often the very existence of the original results. Wielgus and Peebles (2014) reported that the increase in the number of wolves culled the previous year would increase the expected number of livestock killed this year by 4 to 6%. But our results showed that the culling of one wolf the previous year would decrease the expected number of cattle killed this year by 1.9%, and the expected number of sheep killed by 3.4%. However, for every wolf killed there is a corresponding 2.2% increase in the expected number of sheep killed in the same year. The increase in sheep depredation appears to be a short term phenomenon.

## Introduction

There is widespread acceptance that increased lethal control of wolves reduces the number of livestock killed by wolves [1]. Such acceptance has been rarely subject to empirical scrutiny. To the best of Wielgus and Peebles’s [1] knowledge, to which we concur, the long term effectiveness of lethal wolf control–in reducing livestock depredation–has not been “rigorously tested.” In their recent article “Effects of wolf mortality on livestock depredations” published in this journal, Wielgus and Peebles [1] empirically tested the hypothesis that there is a negative relationship between the number of lethally-controlled wolves this year and the number of livestock depredated the following year. Based on statistical modeling, and in contrast to their hypothesis, Wielgus and Peebles [1] reported that the number of livestock depredated the following year was positively associated with the number of wolves killed the previous year. Wielgus and Peebles’s [1] findings are timely given the controversial nature of wolf recolonization associated livestock depredation and the need for effective management and conservation policies. The analysis presented here is additionally motivated by several factors. First, there is nascent evidence that the relationship between the number of wolves killed and the extent of resultant livestock depredation is more complex than examination of only the numbers of killed prey and predators can explain. Factors such as the partial versus complete removal of a wolf pack, wolf social recruitment behaviors, and timing of wolf removal are shown to influence wildlife depredation [2, 3]. Second, Wielgus and Peebles’s [1] implicit call for rigorous testing of the relationship between lethal control of wolves and livestock depredation. The need for rigorous testing, effective management and policy, and our fascination with the counter-intuition of Wielgus and Peebles’s [1] findings led us to scrutinize their analysis, wondering whether the results were artifacts of the analytical procedures used. The results—and validity—of statistical analysis are strongly dependent on the assumptions of the analysis. Certain procedures can substantially distort the results of statistical tests if the assumptions are not met or unmet assumptions are ignored [4]. Thus, to be rigorous and valid, the observational conditions and ensuing data must meet the assumptions of the chosen analytic approach. Unmet assumptions must be accommodated for either by choice of analytic approach or data adjustments.

In the spirit of rigor, validity, and the pressing need for effective management and policy decisions, we proceeded to test the replicability of Wielgus and Peebles’s [1] findings, thanks to the open accessibility of this journal to the requisite data. In science, replication is valuable because no single study can make irrefutable conclusions and progress is made over time through cumulative evidence from several studies [5]. The reliability and validity of a particular scientific finding increases as several independent scholars replicate that specific study and arrive at the same conclusion [6]. Also, the introduction of different analytical procedures to the same data can increase the confirmatory power of empirical findings. Only repeated studies can ascertain the real result from what may be a spurious result from a single study, and safeguard science in the long run [7]. But, despite their merits and attribution of ‘gold standard’, replication studies are scarce in sciences other than the natural sciences [8, 9, 10, 11].

We conducted the replication of Wielgus and Peebles’s [1] study by examining whether there were any oversights in Wielgus and Peebles’s [1] analysis of the data, and reanalyzing the data using the same analytic method but with due attention to the appropriate assumptions and criteria for correct times series analysis [12]. In replicating this study, we show how the omission of minor but important details in the analysis of observational time series data can generate unexpected and misleading results. The results of our replication suggest that Wielgus and Peebles’s [1] best models were misspecified; their conclusions do not hold in the respecified models. We present alternative conclusions given these new findings.

## Methods

Wielgus and Peebles [1] provided the data for all variables used in the analysis in a supplementary table (S1 Table of the original paper). First, we plotted both the cattle and sheep depredation data on the temporal scale. There were long run patterns in all data series and those patterns were persistent in nature (Figures A and B in S1 File). Except for the number of sheep, all other variables display an increasing trend over the years. In such data, we cannot necessarily assume their statistical properties remain constant over time. Modeling these data without appropriate statistical procedures may yield spurious results. If a time-series has an obvious trend, it makes sense to summarize the trend by some simple function of time itself [4, 13]. Furthermore, the temporal dependence in these variables should be assessed and accounted for in the models. Thus to model these data series, trends and lags should be considered in the model selection process [4, 13]. However, we did not find any terms that captured the trends and cycles (apart from taking independent variables from lag periods) in the list of models given in Table 1 and Table 3 or the best models presented in Table 2 and Table 4 by Wielgus and Peebles [1]. We therefore replicated their best models to determine whether the models were correctly specified. The residual plots of the Wielgus and Peebles’s [1] models clearly showed the departure of the residuals from the identically and independently distributed (IID) assumption (Figures A, B, C and D in S2 File), raising legitimate concerns for drawing valid inferences. In time series analysis, the estimator has desired finite sample and asymptotic properties such as unbiasedness and consistency, only when the unexplained residuals are independently and identically distributed. Further statistical tests corroborated this departure because in Wielgus and Peebles’s [1] best models, the assumptions of independence and homogeneity (or stationarity) were violated (p < 0.05). Please see supporting information tables for more information on misspecification tests (Tables A, B and C in S3 File).

For the conditional distribution of livestock depredations given other variables, we followed Wielgus and Peebles [1] in using the negative binomial distribution to build new models. The dependent variables were observed as count data and the variance was much larger than the mean. Therefore, using the same negative binomial distribution for model testing is appropriate. Wielgus and Peebles [1] relied only on AIC and log-likelihood to select the best models. AIC only identifies the best of a set of models that could still be inadequate, and likelihood only generates parameters but does not test inferences. Therefore, AIC and log-likelihood, as measures of goodness-of-fit, are neither necessary nor sufficient for drawing reliable inferences [14]. Moreover, if the model’s assumptions are violated for the data, measures of goodness-of-fit are inferentially irrelevant [14, 15]. Thus, in addition to AIC and log-likelihood, we did misspecification tests to assess the reliability of goodness-of-fit.

For all the models (Wielgus and Peebles’s and re-specified), the same misspecification tests were conducted. Specification errors occur when explanatory variables are correlated with the error term due to various causes such as incorrect functional form, omitted variables, and irrelevant variables in the models. A range of other misspecification tests were also conducted with little difference in the results. Apart from the formal misspecification tests, we evaluated residuals of all the models for their closeness to meeting IID assumption. Our misspecification tests were built to detect heterogeneity, dependence, and extra non-linearity not captured by the null model. In misspecification tests, the statistically significant terms indicate departure from the assumptions of the null models.

In our models, we started with first order lag as done by Wielgus and Peebles [1], and also explored second order lag terms but none of them were statistically significant. We removed the second lag terms in the final models because they did not affect the outcomes of the misspecification tests. We used a linear trend and its removal influenced the misspecification test results, so it was retained in the final models. There was no need for doing even more stringent misspecification tests to arrive at more adequate models because the results were sufficient for a good model as indicated by the p-values [4]. All the estimation procedures were done in R following Venables and Ripley [16].

## Results

### Analytical oversights in the Wielgus and Peebles’s models

There were a couple of analytical oversights in the Wielgus and Peebles’s [1] models. It was surprising not to find any terms in their best models that would account for a trend, given that all the variables had a persistent increasing trend over time. Also, the Wielgus and Peebles’s [1] models should have considered the lag terms of the relevant variables to assess the temporal dependence in the data because last year’s values are more likely to affect this year’s values in this case, for example, the population of wolves produces more wolves through birth and recruitment. It was also quite unusual to have statistically insignificant variables in the best models when the forward selection method was used.

When persistent trends are present in the data, a statistically significant association between the variables could be spurious [17]. Except for the number of sheep, all other variables showed an increasing trend, so the variables might show statistically significant results just because they all followed a common trajectory over time. De-trended data should have been used to make the Figures 1 to 6 in Wielgus and Peebles [1]. Fitting the lines in a bivariate analysis in the presence of trend variation is not appropriate [17]. Also, Wielgus and Peebles [1] did not provide a sound theoretical justification for the choice of quadratic function. One could easily fit cubic or quadratic functions and get completely different results because fitting curves to data and building models to assess uncertainty are different things.

Because the data were not normally distributed, Wielgus and Peebles [1] used negative binomial models. Therefore the Pearson correlation matrix given in Table S2 of the original paper could be misleading. To estimate the Pearson correlation coefficients, the variables should follow the bivariate normal distribution. A major requirement for the bivariate normal distribution is that the variables should have univariate normal distributions. A more important requirement for computing correlations is that the residuals for each data series should be examined separately to verify whether they meet the assumptions of identical and independent distributions. All five variables were time series and there were trends and cycles in the data. At the very least, the trend should have been controlled and non-parametric partial correlation coefficients would have been better [17].

### Cattle killed by wolves

Our results are substantially different from those of Wielgus and Peebles’s [1] (Table 1). In Wielgus and Peebles’s [1] models, the number of wolves killed, the number of wolf breeding pairs, and the number of wolves killed by number of breeding pairs interaction were significant predictors of the number of cattle killed by wolves. Only the number of wolves killed remained significant in our replication; the other two explanatory variables of the original model were not significant. The sign of the number of wolves killed was negative in our case.

**Table 1:** Number of cattle depredated regressed on the number of wolves killed, number of wolf breeding pairs, number of cattle and time index.

Number of cattle depredated (t)	Estimate	Std. Error	z value	Pr (>|z|)	
Time index (trend)	0.16440	0.03102	5.300	< 0.001	***
Number of cattle depredated (t-1)	0.02077	0.00506	4.108	< 0.001	***
Number of wolf breeding pairs (t)	0.02700	0.01563	1.728	0.084	*
Number of wolf breeding pairs (t-1)	-0.02038	0.01650	-1.235	0.217	
Number of wolves killed (t)	0.00802	0.00491	1.634	0.102	
Number of wolves killed (t-1)	-0.01948	0.00527	-3.696	< 0.001	***
Number of cattle (t)	-0.00032	0.00033	-0.950	0.342	
Number of cattle (t-1)	0.00039	0.00033	1.182	0.237	
Intercept	-0.65986	0.55368	-1.192	0.233	

*** significant at the 1% level

** significant at the 5% level

* significant at the 10% level.

AIC = 417.94, 2 Log-likelihood = -397.94, McFadden’s R^2^ = 0.27 (Wielgus and Peebles: AIC = 464.02, 2 Log-likelihood = -452.02, McFadden’s R^2^ = 0.17).

In our model, four explanatory variables were significantly associated with the number of cattle killed by wolves. Although the time index could not be used for substantive explanations, the statistically significant positive coefficient of the trend variable (time index) indicated that there were unobserved factors (or missing explanatory variables) that were associated with the increase in the number of cattle killed by wolves. All being equal, three more cattle were depredated on average from one year to next. In addition, the variables that were statistically controlled in the model also influenced the number of cattle killed by wolves. One wolf killed this year would lead to decrease in the expected number of cattle killed next year by 1.9%. In other words, three wolves culled this year would be associated with one less cattle depredated next year, ceteris paribus. The number of cattle killed the previous year had a statistically significant positive association with the expected number of cattle killed this year. An increase in one breeding pair of wolves was associated with 2.7% increase in the expected number of cattle depredated in the same year. To paraphrase, an increase in two breeding pairs of wolves would lead to one more cattle depredated in the same year, keeping all other factors constant.

### Sheep killed by wolves

When the Wielgus and Peebles’s [1] sheep depredation model was replicated, the results were different in terms of sign, magnitude and significance of the coefficients. The number of statistically significant predictors were fewer than those reported by Wielgus and Peebles (Table 2). In the Wielgus and Peebles’s [1] model, four variables: number of wolves killed, wolf population, number of cattle and number of sheep, and eight interaction terms were significant. Of their four statistically significant variables, only one (number of wolves killed) was statistically significant in our case. The sign of number of wolves killed was negative in this replication.

**Table 2:** Number of sheep depredated regressed on the number of wolves killed, number of wolf breeding pairs, number of sheep and time index.

Number of sheep depredated (t)	Estimate	Std. Error	z value	Pr (>|z|)	
Time index (trend)	0.21481	0.06657	3.227	0.001	***
Number of sheep depredated (t-1)	0.00357	0.00351	1.016	0.310	
Number of wolf breeding pairs (t)	0.03333	0.03227	1.033	0.302	
Number of wolf breeding pairs (t-1)	-0.05263	0.03769	-1.396	0.163	
Number of wolves killed (t)	0.02211	0.00972	2.276	0.023	**
Number of wolves killed (t-1)	-0.03434	0.00930	-3.691	< 0.001	***
Number of sheep (t)	-0.00252	0.00159	-1.585	0.113	
Number of sheep (t-1)	0.00050	0.00148	0.334	0.738	
Intercept	2.04601	1.35157	1.514	0.130	

*** significant at the 1% level

** significant at the 5% level

* significant at the 10% level.

AIC = 506.64, 2 Log-likelihood = -486.64, McFadden’s R^2^ = 0.18 (Wielgus and Peebles: AIC = 544.04, 2 Log-likelihood = -510.05, McFadden’s R^2^ = 0.14).

In our model, the statistically significant positive coefficient of the trend variable meant that there was a long run increasing trend in the number of sheep killed by wolves which was not explained by the explanatory variables included in the model. From one year to next, six more sheep were depredated on average when the influences of other factors are controlled. A unit increase in the number of wolves culled this year would reduce the expected number of sheep killed by 3.4% next year. Ceteris paribus, one more wolf killed this year would meant one less sheep depredated next year. For every wolf killed, there was a corresponding 2.2% increase in the expected number of sheep killed in the same year. In other words, there was an increase in the depredation by one sheep for every two wolves culled in the same year, all things being equal.

## Discussion

We did not find any statistical support for the Wielgus and Peebles’s [1] findings in this replication. This could be because the original models were misspecified. Rather than more culling of wolves leading to more killings of livestock in the following year, our results indicate that more culling of wolves would lead to fewer killings of livestock in the following year than expected in the absence of culling. Two recent studies conducted at the wolf pack level also support our findings directly or indirectly. In the same study area, Bradley and others [3] report that compared to no removal, partial and full pack removal of wolves reduced the occurrence of subsequent livestock depredations by 29% and 79%, respectively, over a span of 5 years. Similarly in Idaho, it was found that killing of wolves would lead to decline in the recruitment (as measured by pup survival to 15 months) in wolf populations [2], which may in turn lead to fewer livestock depredations in subsequent years. How wolf populations respond to lethal control is a complex phenomenon. It seems that wolf removal reduces livestock depredations but the magnitude somewhat depends on the type and timing of removal and timing, as well as the recruitment behaviors of wolves.

Also, there is a positive association between the number of wolves killed and the expected number of livestock killed in the same year. This association does not necessarily negate the premise that wolves killed may lead to “fracture of pack structure and increased breeding pairs” which eventually lead to increased livestock depredation [18]. Also, the positive association between the number of wolves and the expected number of livestock killed in the same year does not negate the social disruption hypothesis [19]. However, there is an inherent disconnect between the biological scale of data (statewide) used by Wielgus and Peebles [1] and the biological scale of the social disruption hypothesis (the individual pack). While such direct effect may exist, the correlational nature of the data and discrepancy of scale don’t really make hypothesis directly testable. The rather brief nature of the effect (same year and with a reverse effect the following year) suggests that the effects of fracture of pack structure and other social disruptions are rather short term phenomena. According to our findings, it is possible that the reorganization of pack structure and consequent reduction in breeding pairs, as well as the emergence of a new social order in disrupted packs may occur by the following year. Additionally, and given the observational nature of the study, it is not clear whether more killings of livestock draws the attention to kill more wolves or wolves go on rampant killing of livestock when they are hunted more frequently. Either way, caution should be exercised regarding the potential impact of wolf culling on livestock depredation, especially in the long term. These interpretations are further supported by the findings of Ausband et al. [2] and Bradley et al. [3].

The failure of our attempt to reproduce the results using the same data and following more rigorous statistical procedures casts doubt on the reliability and validity of the conclusions drawn from the Wielgus and Peebles’s [1] models. If various models with their differing assumptions lead to similar results, the finding is robust and we have greater confidence on the finding [20]. Almost all statistical procedures have assumptions, and the results yielding from those procedures are valid and reliable only if the underlying assumptions are met. This replication study also highlights the need for testing the assumptions in empirical modeling. Had some information regarding the diagnostic tests been reported by Wielgus and Peebles [1], such as a graph of residuals, it would have been easier to detect the analytical oversights.

The open access to the data policy of this journal made it easy to replicate the study. Providing the data publicly saved time and effort to acquire the data and also served as a major impetus to replicate the study. Had these ‘counter-intuitive’ findings not been scrutinized closely through replication, the accidental findings would have been propagated as general truth. Any management and conservation practices and decisions informed by such accidental findings can be problematic.

The nature of data itself pose some major limitations for analysis. Based on wolf ecology, wolf packs appear to be most appropriate unit of analysis to test the hypotheses [18]. Both wolf population and livestock depredation data are grouped together (aggregated) at the state level. When the data from different wolf packs are combined, the inferential statistics (such as correlation or regression coefficients) for the resultant data set may not characterize the relationship between X and Y in any of the constituent packs. This problem occurs because the aggregate statistics reflect not only the relationship between X and Y within different wolf packs, but also the differences among the group means [21]. We therefore recommend replication of the study with data analysis done at the wolf pack level. Such an effort would be helpful to test the robustness of our findings.

## Supporting Information

S1 File Temporal variation in livestock killed. (DOCX)

S2 File Residual plots of the models. (DOCX)

S3 File Misspecification tests. (DOCX)

S4 File R codes used in replication. (TXT)

S5 File Data. (XLS)
