# Supplementary material for: Wolf Lethal Control and Livestock Depredations: Counter-Evidence from Respecified Models
Source: PLoS One. 2016 Feb 11;11(2):e0148743. doi: 10.1371/journal.pone.0148743 (PMC4751083; doi:10.1371/journal.pone.0148743)

**S1**: Temporal variation in livestock killed

Fig A: An increasing trend in the number of cattle killed by wolves in a year in Wyoming, Montana and Idaho states from 1988 to 2012. There is a variation across years and among states.


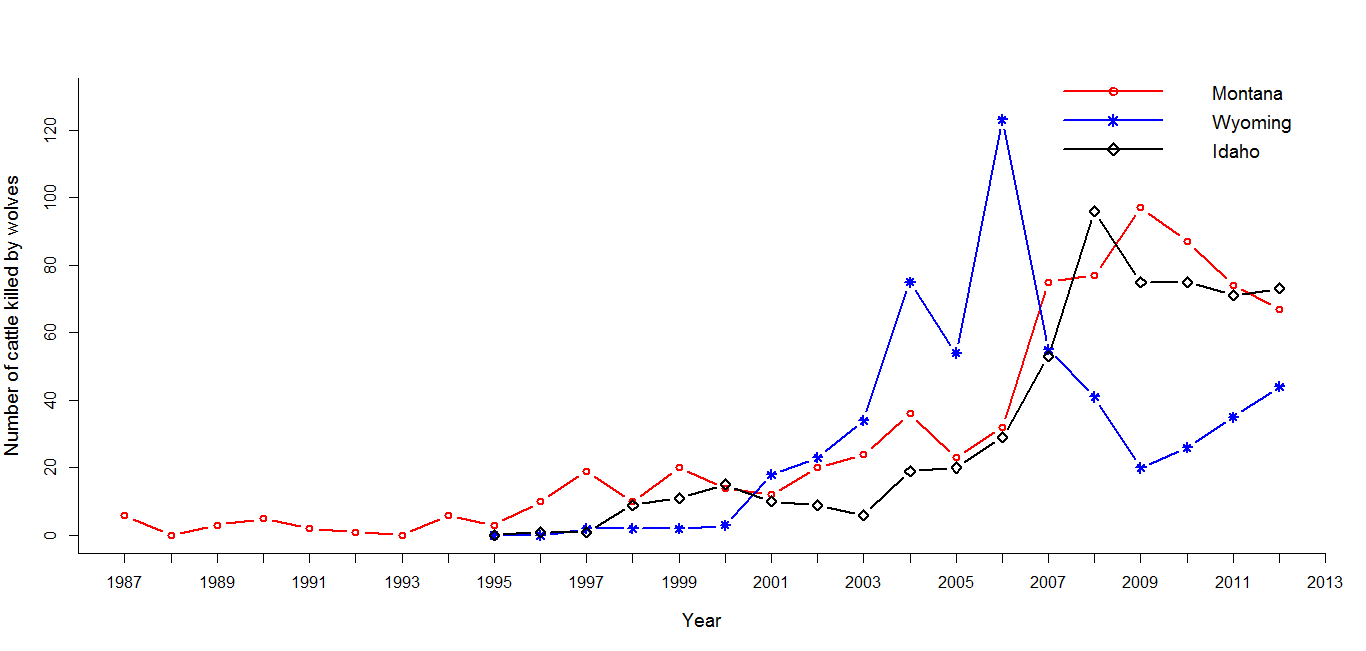


Fig B: An increasing trend in the number of sheep killed by wolves in a year in Wyoming, Montana and Idaho states from 1988 to 2012. There is a great deal of variation across years and among states.


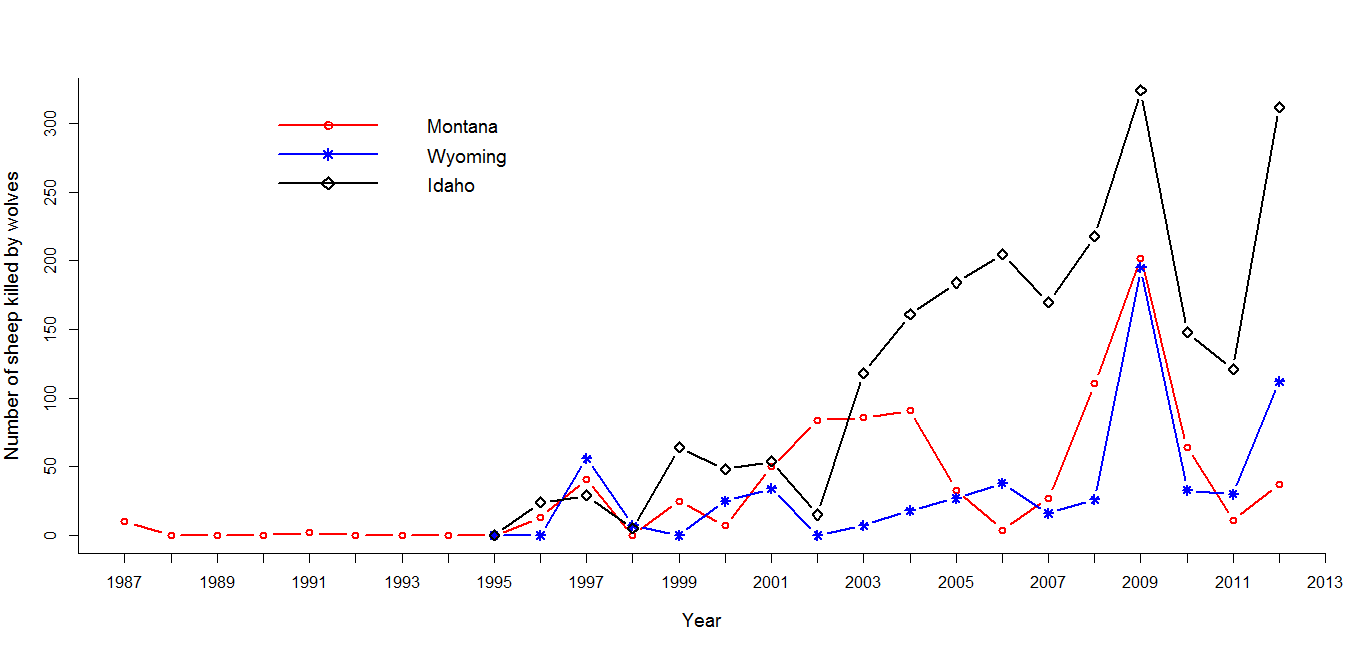

Supplement: S1 File — (DOCX) [file pone.0148743.s001.docx]
