# Supplementary material for: Wolf Lethal Control and Livestock Depredations: Counter-Evidence from Respecified Models
Source: PLoS One. 2016 Feb 11;11(2):e0148743. doi: 10.1371/journal.pone.0148743 (PMC4751083; doi:10.1371/journal.pone.0148743)

**S2**: Residual plots of the models

Fig A: In the residuals of the Wielgus and Peebles’ model of the number of cattle depredated by wolves, there is still some systematic component not accounted by the model.


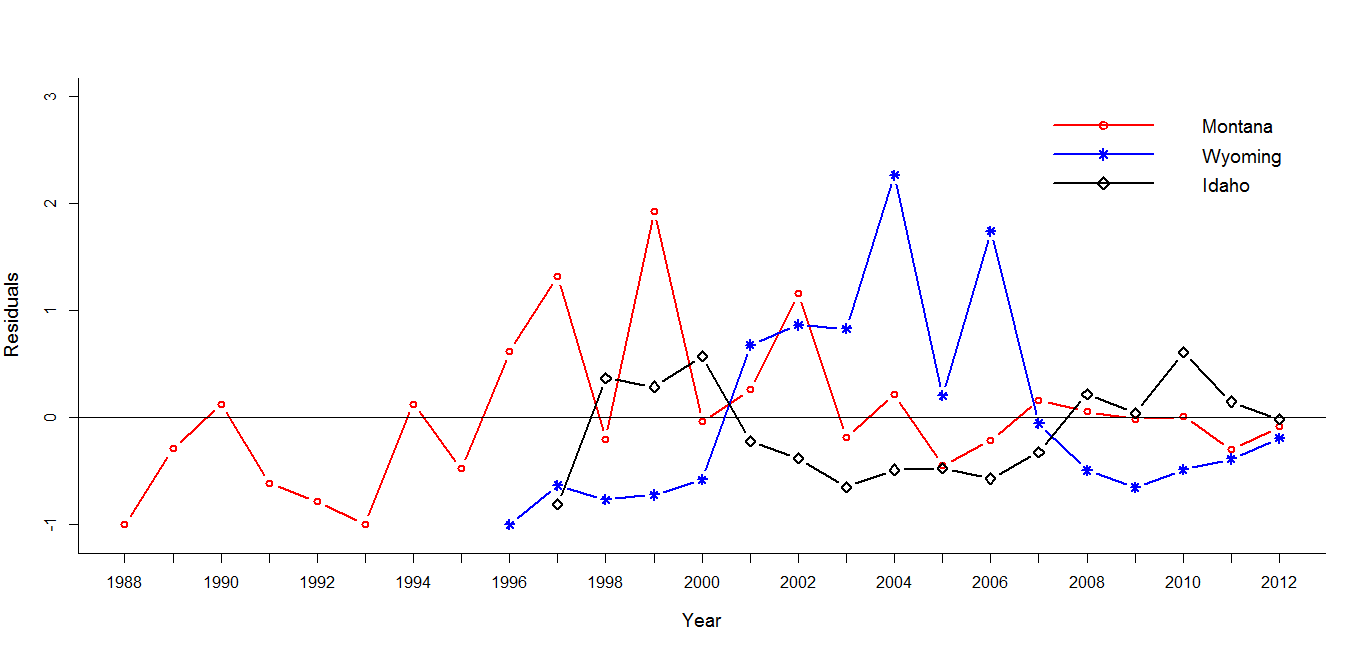


Fig B: There is almost no systematic component in the residuals of the correctly specified model of the number of cattle depredated by wolves.


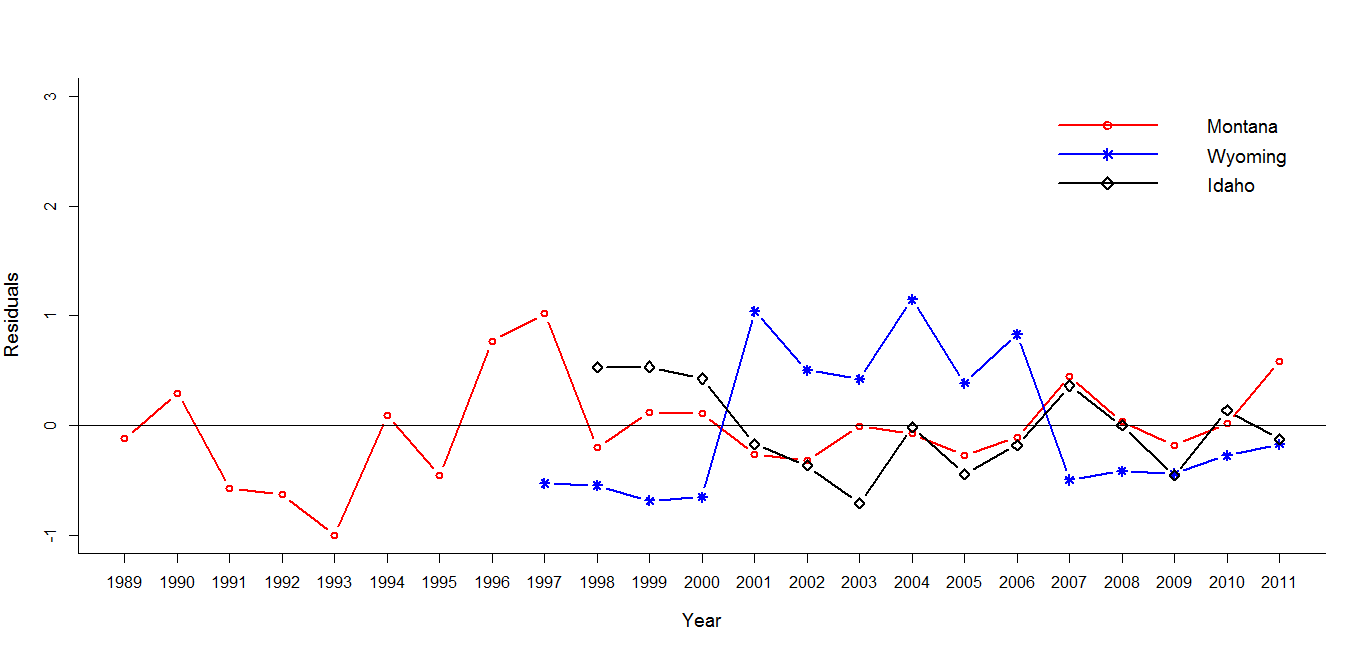


Fig C: The residuals of the Wielgus and Peebles’ model of the number of sheep killed by wolves.


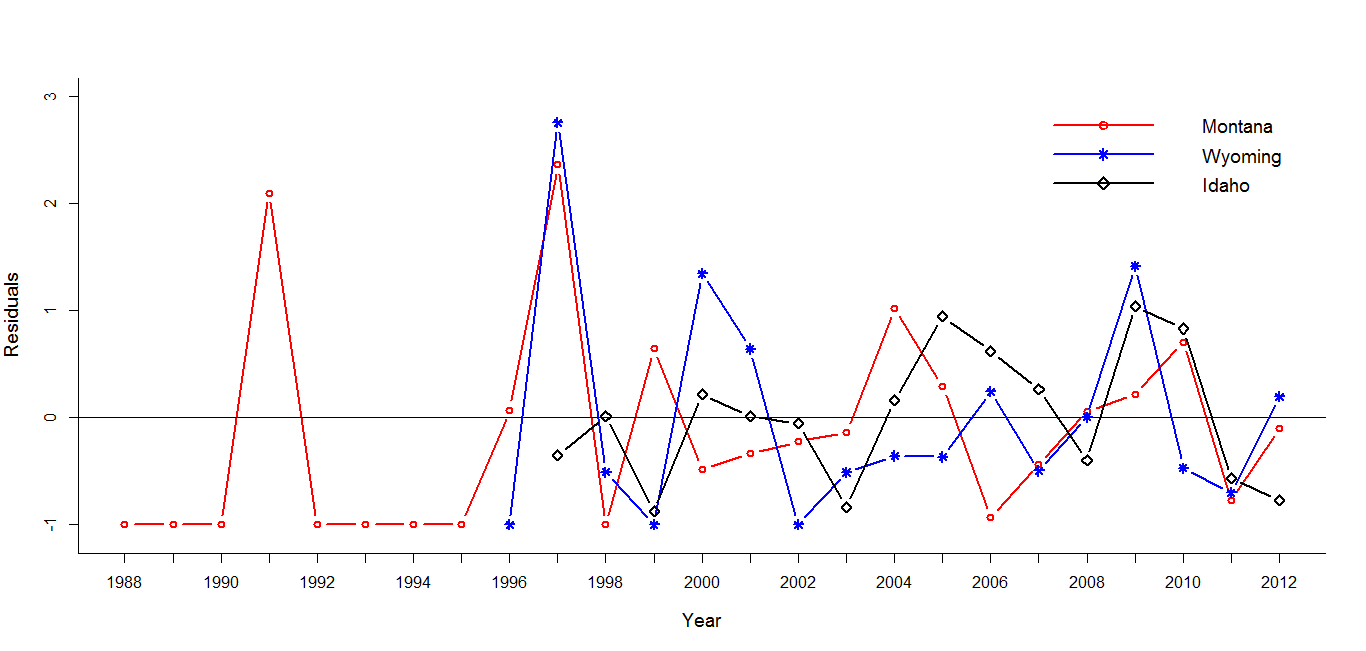


Fig D: The residuals of the correctly specified model of the number of sheep killed by wolves.


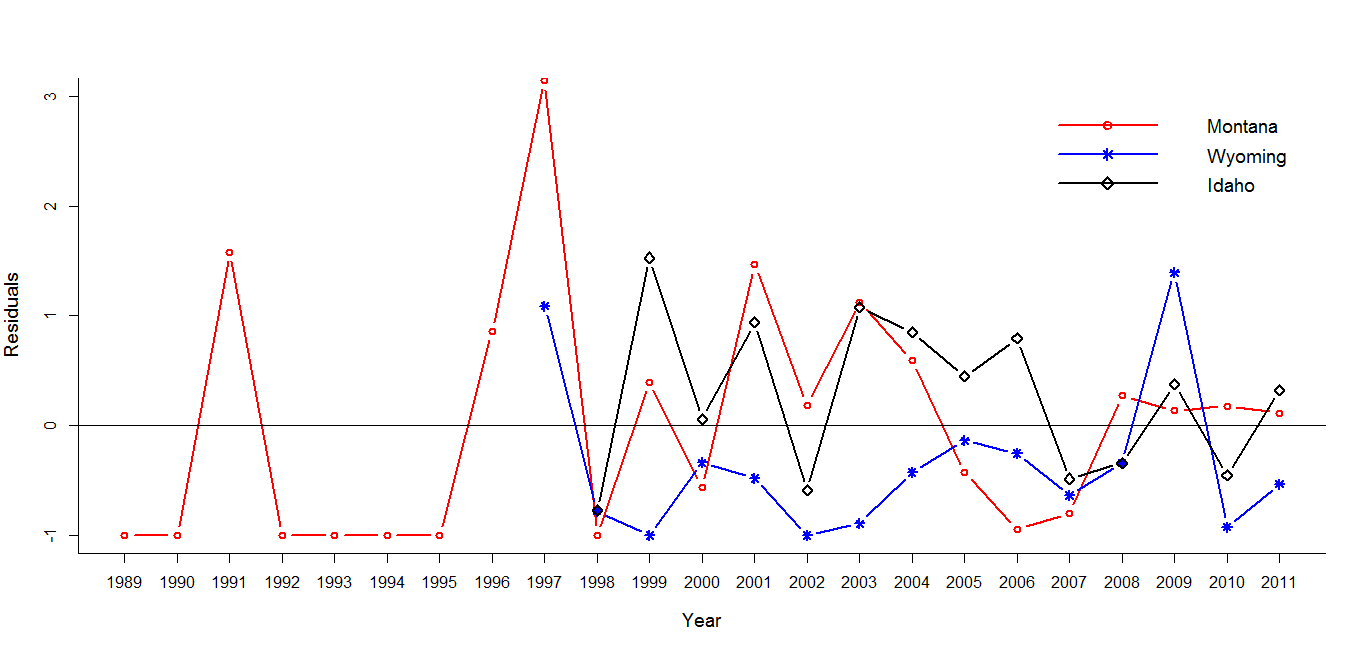

Supplement: S2 File — (DOCX) [file pone.0148743.s002.docx]
