# Supplementary material for: Wolf Lethal Control and Livestock Depredations: Counter-Evidence from Respecified Models
Source: PLoS One. 2016 Feb 11;11(2):e0148743. doi: 10.1371/journal.pone.0148743 (PMC4751083; doi:10.1371/journal.pone.0148743)
Supplement: S3 File — (DOCX) [file pone.0148743.s003.docx]

**S3** Misspecification tests

For the misspecification test, we used the following auxiliary regression. The explanatory terms are supposed to capture any departure from the null hypothesis of model being correctly specified.

$$\hat{u}_{it}=\alpha_{0}+\alpha_{1}\hat{y}_{it}+\alpha_{2}\hat{y}_{it-1}+\alpha_{3}{\hat{y}_{it}}^{2}+\alpha_{4}t+\alpha_{5}t^{2}$$

$\hat{u}_{it}$ = residuals of the model

$\hat{y}_{it}$ = fitted value of the model (represents all the terms included in the null model)

$\hat{y}_{it-1},{\hat{y}_{it}}^{2},t,t^{2}$= terms that are expected to capture any departure from the null if left unaccounted for.

Table A: Null hypotheses to test the three major assumptions for statistical modeling.

| Tests to assess for | Null Hypothesis |
| --- | --- |
| Dependence | α_2_ = 0 |
| Non-linearity | α_3_ = 0 |
| Homogeneity (stationarity) | α_4_ = 0, α_5_ = 0 |

OLS was used to estimate the above auxiliary regression. The same auxiliary regression was used to conduct all the misspecification (M-S) tests and the results are summarized in the following tables.

Table B: Results of the misspecification tests of the Wielgus and Peebles’ model and re-specified model of cattle depredation. The significant coefficient (α_4_) indicates that the assumption of homogeneity was violated in the Wielgus and Peebles’ model. All insignificant coefficients indicate that the assumptions are met in the re-specified model.

|  | Wielgus and Peebles’ Model | | Re-specified Model | |
| --- | --- | --- | --- | --- |
| Coefficient | Estimate | p - value | Estimate | p - value |
| α_0_ | -0.873 | 0.081 | -0.302 | 0.213 |
| α_1_ | -0.025 | 0.199 | 0.011 | 0.254 |
| α_2_ | 0.005 | 0.602 | -0.002 | 0.597 |
| α_3_ | 0.000 | 0.470 | 0.000 | 0.184 |
| α_4_ | **0.147** | **0.057** | 0.018 | 0.424 |
| α_5_ | -0.003 | 0.287 | 0.000 | 0.382 |

Table C: Results of the misspecification tests of the sheep depredation models. The insignificant coefficients indicate that the assumptions are met.

|  | Wielgus and Peebles’ Model | | Re-specified Model | |
| --- | --- | --- | --- | --- |
| Coefficient | Estimate | p - value | Estimate | p - value |
| α_0_ | 0.613 | 0.329 | 0.914 | 0.203 |
| α_1_ | < 0.001 | 0.999 | -0.003 | 0.373 |
| α_2_ | 0.003 | 0.207 | 0.001 | 0.452 |
| α_3_ | < 0.001 | 0.454 | < 0.001 | 0.625 |
| α_4_ | 0.084 | 0.377 | 0.149 | 0.169 |
| α_5_ | 0.003 | 0.363 | 0.004 | 0.241 |
